# Effects of Plateau Pika Disturbance on the Spatial Heterogeneity of Vegetation in Alpine Meadows

**DOI:** 10.3389/fpls.2021.771058

**Published:** 2021-11-03

**Authors:** Jie Li, Hao Hao Qi, Yuan Yuan Duan, Zheng Gang Guo

**Affiliations:** State Key Laboratory of Grassland Agro-Ecosystems, Key Laboratory of Grassland Livestock Industry Innovation, Ministry of Agriculture and Rural Affairs, Engineering Research Center of Grassland Industry, Ministry of Education, College of Pastoral Agriculture Science and Technology, Lanzhou University, Lanzhou, China

**Keywords:** *Ochotona curzoniae*, disturbance intensity, spatial heterogeneity of vegetation, Qinghai-Tibetan Plateau, alpine meadows

## Abstract

Herbivory is one of the important factors that influence spatial heterogeneity of vegetation in grasslands. In this study, we focused on plateau pika (*Ochotona curzoniae*) to investigate the effects of the presence of small semi-fossorial herbivores and their disturbance intensity on the spatial heterogeneity of vegetation in alpine meadows across three sites in the Qinghai-Tibetan Plateau. A random stratified paired sampling method was used to collect vegetation data, and plant species richness at both fine and coarse scales were used to estimate the spatial heterogeneity of vegetation. This study showed that the presence of plateau pikas led to higher spatial heterogeneity of vegetation in alpine meadows, which increased linearly as the disturbance intensities of plateau pikas increased. The findings of this study demonstrate that small semi-fossorial herbivores have an important impact on the spatial heterogeneity of vegetation, and present a possible approach for estimating the effect of the presence of a small semi-fossorial herbivore and its disturbance intensity on the spatial heterogeneity of vegetation in grasslands.

## Introduction

Spatial heterogeneity of vegetation is an important proxy for gaining insight into grassland plant productivity since it is usually positive related to plant productivity in grasslands (Bai et al., 2007; Otieno et al., 2011; Song et al., 2020). The spatial heterogeneity of vegetation is usually estimated by non-spatial statistics and spatially explicit metrics (Kolasa and Rollo, 1991; Adler et al., 2001). In contrast to non-spatial statistics, spatially explicit metrics, containing many parameters of grassland plant communities (Bangert and Slobodchikoff, 2006; Bloor et al., 2020), are considered to predict the spatial heterogeneity of vegetation because a parameter value of grassland plant communities can be used to reasonably predict another parameter value in the same area (Adler et al., 2001). Although there are many parameters in spatially explicit metrics to estimate spatial heterogeneity of vegetation in grasslands, plant species richness is widely applied to estimate the spatial heterogeneity of vegetation in grasslands (Questad and Foster, 2007; Case et al., 2013; Lindtner et al., 2020). The plant species richness is closely related to the plant community structure and community productivity in grasslands (Hector et al., 1999; Tilman et al., 2006; Pang et al., 2021) and varies at different spatial scales in the field (Dumbrell et al., 2008). However, plant species richness is affected by biotic factors (Adler and Lauenroth, 2000; Altesor et al., 2005; Pang et al., 2021), which have an important impact on the spatial heterogeneity of vegetation in grasslands (Jones et al., 2008; Yoshihara et al., 2009).

Herbivores are considered as important biotic factors that influence the spatial heterogeneity of vegetation in grasslands (Jones et al., 1997; Adler and Lauenroth, 2000; Davidson et al., 2012; Wei et al., 2019). Large herbivore grazing has been verified to increase the spatial heterogeneity of vegetation in grasslands (Adler and Lauenroth, 2000; Adler et al., 2001; Song et al., 2020; Michaels et al., 2021). In addition to large herbivores, many kinds of small semi-fossorial herbivores inhabit grasslands (Bagchi et al., 2006; Questad and Foster, 2007; Lindtner et al., 2020; Wei et al., 2020) and these small semi-fossorial herbivores often cause extensive disturbance to grassland vegetation and soil (Hobbs and Huenneke, 1992; Liu et al., 2017; Smith et al., 2019). They often modify the interspecies relationships among plants through selective feeding behavior and induce several fertilizer islands through redistribution of soil nutrients (Case et al., 2013; Liu et al., 2013; Yurkewycz et al., 2014; Yu et al., 2017). This enables some plants to reconstruct their niche (Jones et al., 2008), and consequently develop new and different plant assemblages (Milton et al., 1997; Questad and Foster, 2007; Scherrer et al., 2019), compared to areas where small semi-fossorial herbivores are absent. Thus, small semi-fossorial herbivores often lead to differences in grassland vegetation based on the presence/absence of semi-fossorial herbivores (Wilson and Smith, 2015; Wang et al., 2018). Lindtner et al. (2020) used plant species richness at fine scale (average species number of many subplots with the size of 1 × 1 m) and coarse scales (total number of plant species in that subplots) to estimate the spatial heterogeneity of vegetation in grasslands, and found that the spatial heterogeneity of vegetation in grasslands increased with increasing disturbance intensity of European ground squirrel (*Spermophilus citellus*) in the United States. However, this study did not use grasslands without European ground squirrel as a reference area for comparison. Further, whether the presence of small semi-fossorial influences the spatial heterogeneity of vegetation in grasslands is not well documented. Therefore, more studies are needed to investigate the effect of small semi-fossorial herbivores on the spatial heterogeneity of vegetation in grasslands, which can determine the relationship between small semi-fossorial herbivores and the spatial heterogeneity of vegetation in grasslands.

Plateau pika (*Ochotona curzoniae*) is a small, social semi-fossorial herbivore native to alpine grasslands in Asia (Fan et al., 1999; Davidson et al., 2012), particularly in the alpine meadows on the Qinghai-Tibetan Plateau (Smith et al., 2019). This small semi-fossorial herbivore often disturbs the alpine meadow through excretion of feces and urine (Fan et al., 1999), selectively consuming plants (Liu et al., 2013), producing bare soil patches (Pang et al., 2020), and aerating the soil (Wilson and Smith, 2015; Yu et al., 2017; Pang et al., 2021). This often leads to discrete mosaics of vegetated surfaces and bare soil patches over a range of different spatial scales in the alpine meadows (Wilson and Smith, 2015; Zhao et al., 2021). At a large spatial scale, the plateau pikas are distributed territorially and patchily on alpine meadows (Pang et al., 2020), because they prefer to live in low and open habitats to avoid predators (Fan et al., 1999; Zhang et al., 2020). Once the plateau pikas occupy a given suitable area, their activities often enable alpine meadows in that area to further degrade (Liu et al., 2013). In reality, some alpine meadows are low and open and are a potential habitat for the species, but are not inhabited by them (Fan et al., 1999), because the diffusion of plateau pikas is a gradual process (Wang et al., 2020). Thus, selecting the potential habitats for plateau pikas as reference areas is a possible way to test whether the presence of plateau pikas influences the spatial heterogeneity of vegetation in grasslands.

Plateau pikas have been found to live in various habitats with different soil types, topographies, microclimates, and vegetation types on the Qinghai-Tibetan Plateau (Smith and Foggin, 1999; Guo et al., 2012). If field surveys are conducted at only one site with similar environmental conditions, there will be uncertainty regarding the changes in the spatial heterogeneity of vegetation resulting from the presence of plateau pikas singly or in combination with environmental factors. Thus, multiple sites are necessary to identify the general pattern of the presence of plateau pikas influencing the spatial heterogeneity of vegetation in alpine meadows. In this study, we used the plateau pika as an example study animal to examine the effect of the presence of small semi-fossorial herbivores on the spatial heterogeneity of vegetation in grasslands across three sites. Specifically, this study hypothesized that: (1) the presence of plateau pikas will lead to higher spatial heterogeneity of vegetation in grasslands, and (2) the spatial heterogeneity of vegetation in grasslands will increase as the disturbance intensity of plateau pikas increases, which will provide useful information for quantify the relationship between small semi-fossorial herbivores and grassland plant productivity.

## Materials and Methods

### Study Site Description

**FIGURE 1:**
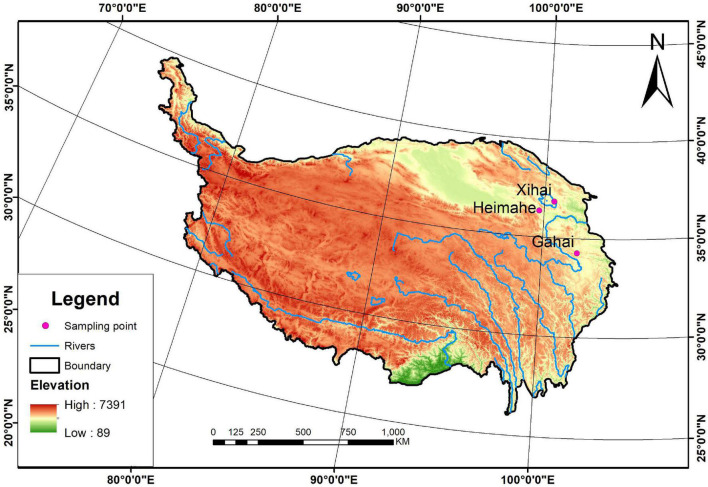
The three survey sites in the Qinghai-Tibetan Plateau.

Three survey sites with different environmental conditions were selected to examine the effects of plateau pikas and their disturbance intensity on the spatial heterogeneity of vegetation in alpine grasslands on the Qinghai-Tibetan Plateau. These sites were located at Heimahe (99°41′ E, 36°21′ N) in Gonghe County, Xihai Town (100°37′ E, 36°51′ N) in Haiyan County, Qinghai Province, and at Gahai town (102°10′ E, 34°16′ N) in Luqu County, Gansu province (Figure 1). The three survey sites range in elevation from 3,000 to 4,650 m and in average annual precipitation from 250 to 800 mm, and experience a similar cold, humid continental plateau climate (Table 1). The soils at the three survey sites were classified into alpine meadow soils based on the Chinese Soil Classification System, similar to Cambisols in the world reference base (WRB) soil classification system. The main alpine grasslands in the study site are alpine meadows dominated by sedges (Liu et al., 2017), and these alpine meadows have been contracted to farm households. Each household has managed alpine meadows by classifying it into warm grazing areas and cold grazing areas; cold grazing areas were fenced from mid-April to early October and the fence was opened to yaks and Tibetan sheep for grazing from mid-October to early April in the last few decades. The plateau pika are the only small semi-fossorial herbivores at the surveyed sites, although there are many small herbivores throughout the counties.

**TABLE 1:** Description of the study areas at Gonghe, Haiyan, and Luqu.

Location	Elevation (m)	MAT (°C)	MAP (mm)	Dominant species	Main associate species
Gonghe	3,750	4.1	250–500	*Kobresia pygmaea*	*Elymus nutans*
					*Poa pratensis*
					*Anemone obtusiloba*
Haiyan	3,270	1.5	300–500	*K. humilis*	*P. pratensis*
					*Leontopodium nanum*
					*Potentilla bifurca*
Luqu	3,550	2.3	600–800	*K. pygmaea*	*E. nutans*
					*P. pratensis*
					*A. obtusiloba*

*MAT is the mean annual temperature; MAP is the mean annual precipitation.*

### Experimental Design

The survey areas at each site were selected in the cold grazing areas that were relatively flat. In this study, a random stratified and paired design was used to select the plots. At each survey area, we randomly selected the first disturbed plot where plateau pikas were visible or the active burrow entrances were found, and then selected the second disturbed plot along the road. The distance between two disturbed plots was greater than 3 km. In a similar fashion, 10 disturbed plots were finally selected at each survey site. Because the average area of the plateau pika’s home range has been reported as 1,262.5 m^2^, the plot size was designed as 35 m × 35 m, which was approximately equivalent to the home range (Fan et al., 1999). Following this, a paired adjacent undisturbed plot, without visible plateau pikas or active burrow entrance, was selected for each disturbed plot. To ensure that the undisturbed plot was a true reference area, the distance between the paired plots was maintained at 500–1,000 m, which ensures avoiding the overlap between disturbed and undisturbed plots. To ensure that each disturbed plot had a paired undisturbed plot to the extent possible, each paired plot was placed on the same alpine meadow, which ensured that each paired plot had no obvious differences in soil type, topography, microclimate, and vegetation composition. In total, this study surveyed 30 paired plots across three sites, consisting of 30 each of disturbed and undisturbed plots. Each paired plot was managed as a single unit. The active burrow entrances at each disturbed plot were used to estimate the disturbance intensity of the plateau pikas. The disturbance intensity of plateau pikas was possibly different among the 30 disturbed plots, which was helpful for identifying a general pattern of plateau pika disturbance in relation to the spatial heterogeneity of vegetation.

### Field Survey and Sampling

Plateau pikas disturb alpine meadows mostly in August (Wang et al., 2018), and field surveys were conducted in early August 2020. First, the active burrow entrance at each disturbed plot was estimated by the “plugging tunnels method,” in which the burrow entrances were plugged with dry hay for 3 days, and the number of plugs that were cleared by the plateau pikas to allow access to the meadow surface was recorded (Zhang et al., 2020). The average number of burrow entrances with cleared plugs in 3 days was considered as the disturbance density of plateau pikas. Second, five subplots with a W pattern were arranged in each plot. The size of the subplot was 1 × 1 m and the distance between the two subplots was approximately 8 m. The subplots in the disturbed plots were shifted slightly to avoid bare soil patches, if needed. All vascular plant species in each subplot were identified.

### Estimation of Spatial Heterogeneity of Vegetation

Lindtner et al. (2020) proposed that the spatial heterogeneity of vegetation was estimated by β = γ/α*-1*, where β represents the spatial heterogeneity of vegetation, γ represents the plant species richness at a coarse scale, and α is the plant species richness at a fine scale. The plant species richness at fine scale was calculated by the average value of five subplots in a plot, and the plant species richness at coarse scale was calculated by the total number of plant species in all five subplots of a plot.

### Statistical Analysis

All analyses were performed using R version 4.0.2 (R Core Team, 2020). A linear mixed model (LMM) with the function “lmer” from the lme4 package was used to examine the effects of the presence of plateau pikas on the spatial heterogeneity of vegetation across the three sites, in which paired design nested within each site (proceeding nested design analysis) was included as a random factor. To test the general pattern, a paired-samples *t*-test was used to examine the effect of the presence of plateau pikas on the spatial heterogeneity of vegetation at each site.

To clarify the responses of spatial heterogeneity of vegetation to the disturbance intensity of plateau pikas, a linear model (LM) was used to construct the regression models, describing relationships of spatial heterogeneity of vegetation with disturbance intensity of plateau pikas, in which the disturbance intensity of plateau pikas was considered to be the fixed factor.

## Results

### Effect of the Presence of Plateau Pikas on Spatial Heterogeneity of Vegetation Resource

**FIGURE 2:**
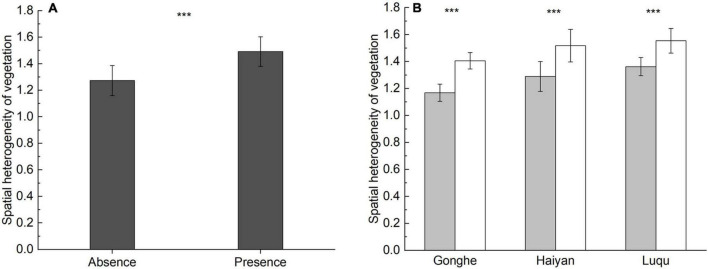
Spatial heterogeneity of vegetation (mean ± standard error) in the presence or absence of plateau pika when the data from three sites was analyzed together **(A)** and in each site **(B)**. The statistics were based on the generalized linear mixed models (GLMMs) and the paired-samples *t*-test, with the paired plots nested within sites as random factors. “***” indicates significant differences at *P* < 0.001.

Accounting for an overall scale effect, the spatial heterogeneity of vegetation was higher in the presence of plateau pikas than in their absence (spatial heterogeneity of vegetation: *F* = 139.198, *P* < 0.001) (Figure 2). Accounting for individual site effects, the spatial heterogeneity of vegetation in the presence of plateau pikas was higher than that in their absence at each site, similar to the results from the three sites together. These results presented a general pattern regarding the spatial heterogeneity of vegetation in relation to the presence of plateau pikas.

### Effect of Disturbance Intensity of Plateau Pikas on Spatial Heterogeneity of Vegetation

**FIGURE 3:**
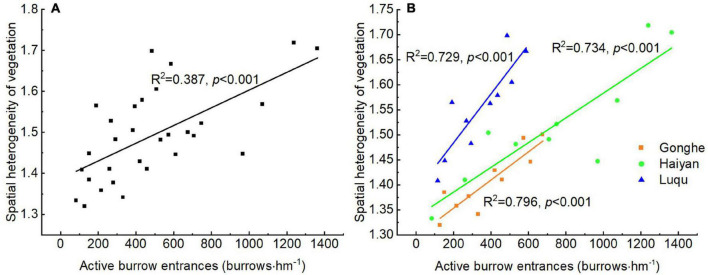
Relationship between spatial heterogeneity of vegetation and the disturbance intensity of plateau pikas across three sites **(A)** and in each site **(B)** based on linear models (LMs).

An analysis of the data from the three sites or an individual site suggested that there was a linear increase in the spatial heterogeneity of vegetation with the increase in disturbance intensity of plateau pikas (Figure 3). This indicated that there was a general pattern of disturbance intensity of plateau pikas influencing the spatial heterogeneity of vegetation.

## Discussion

Herbivory is one of the important biotic factors that induce the spatial heterogeneity of vegetation in grasslands (Adler and Lauenroth, 2000; Adler et al., 2001; Bangert and Slobodchikoff, 2006; Case et al., 2013), and optimal spatial heterogeneity of vegetation is acknowledged as an effective approach to maintain grassland ecosystem functions such as plant species richness and productivity (Jones et al., 1997; Bakker et al., 2006; Davidson et al., 2012; Song et al., 2020). Previous studies have shown that the spatial heterogeneity of vegetation is different between fine and coarse scales in the presence of small semi-fossorial herbivores, such as prairie voles (*Microtus ochrogaster*) (Questad and Foster, 2007) and Siberian marmots (*Marmota sibirica*) (Yoshihara et al., 2009), whereas these studies did not consider the areas without these animals as true reference areas for comparison. In this study, we select areas without plateau pikas as reference areas to examine the effect of the presence of plateau pikas on the spatial heterogeneity of vegetation across three sites, in an attempt to identify a general pattern concerning the spatial heterogeneity of vegetation in relation to the presence of a small semi-fossorial herbivore and its disturbance intensity.

We found a higher spatial heterogeneity of vegetation in the presence of plateau pikas than in their absence, which indicates that the presence of plateau pikas leads to higher spatial heterogeneity of vegetation, in agreement with the first hypothesis. The higher spatial heterogeneity of vegetation is caused by the following reasons. First, plateau pikas prefer to clip tall plants near the active burrow entrance to monitor predators (Liu et al., 2009; Zhang et al., 2020), which increases light availability for short plant species and rare plants (Borer et al., 2014; Zhang et al., 2020), and forms new and various plant assemblages near the active burrow entrance, resulting in more spatial heterogeneity of vegetation at multiple spatial scales (Milton et al., 1997; Questad and Foster, 2007). Second, plateau pikas prefer graminoids as food throughout their home range (Liu et al., 2017), which alters the existing interspecific and intraspecific relationships among plants because graminoids are often dominant plants (Pang and Guo, 2018). This usually divides the areas with plateau pikas into alpine meadows dominated by strongly competitive dominant species and other alpine meadows dominated by disturbance-dependent species (Yoshihara et al., 2009; Kelemen et al., 2019), contributing to an increase in spatial heterogeneity of vegetation. Third, the presence of plateau pikas is beneficial to seed dispersal (Dobson et al., 1998). Some dispersed species cannot survive in a given habitat due to their weak competitive ability (Zhang et al., 2020), while others can survive in that habitat when they disperse and colonize (Wang et al., 2018, 2020), which develops more different plant communities in the presence of plateau pikas, increasing the spatial heterogeneity of vegetation. Fourth, plateau pikas often create a “spatiotemporal mosaic” of vegetated surface and bare soil patches (Pang et al., 2020), and these soil patches can provide suitable habitats for seeds of opportunistic plants for emergence and colonization (Bochet, 2015). The bare soil patches are similar to mounds created by small subterranean mammals, which can further increase the spatial heterogeneity of vegetation (Jones et al., 2008; Case et al., 2013). These four processes result in a higher spatial heterogeneity of vegetation in the presence of plateau pikas, indicating that the presence of plateau pikas is beneficial to plant productivity in alpine meadows since the spatial heterogeneity of vegetation is positive related to grassland plant productivity (Bai et al., 2007; Otieno et al., 2011; Song et al., 2020).

This study also found that the spatial heterogeneity of vegetation showed a linear increasing trend as the disturbance intensity of plateau pikas increased, which is consistent with the second hypothesis. This is similar to the relationships recorded between the spatial heterogeneity of vegetation and the disturbance intensity of European ground squirrels (Lindtner et al., 2020). In this case, three mechanisms can be used to explain the spatial heterogeneity of vegetation in relation to the disturbance intensity of plateau pikas. First, clipping behavior by plateau pikas near active burrow entrance increases as the disturbance intensity of plateau pikas increases (Liu et al., 2009). This provides more selectivity for the colonization of different species and develops more plant assemblages with unique features, contributing to an increase in spatial heterogeneity of vegetation. Second, more bare soil patches are accompanied by an increase in disturbance intensity of plateau pikas (Yu et al., 2017; Smith et al., 2019; Pang et al., 2020), which forms more diverse fertilizer islands and provides favorable conditions for the invasion and colonization of pioneer species, resulting in more diversification of species assemblages and an increase in spatial heterogeneity of vegetation. Third, graminoids consumed by plateau pikas become more frequent as the disturbance intensity of plateau pikas increases (Pang and Guo, 2017; Wei et al., 2020), which further decreases the dominance degree of dominant graminoids in the plateau pika home range. This leads to reconstruction of plants in the plateau pika home range, forming more unique plant communities at the microhabitat scale (Scherrer et al., 2019), contributing to an increase in the spatial heterogeneity of vegetation in the plateau pika home range (Yoshihara et al., 2009), which implies that the plant productivity in alpine meadows can increase as the disturbance intensity of plateau pikas increase. Although 30 disturbed plots, containing different disturbance intensity, is used to identify the general pattern concerning the effect of disturbance intensities on spatial heterogeneity of vegetation, some previous studies have verified that higher disturbance intensity of plateau pikas often results in higher unpalatable plant biomass (mainly consisting of forbs) and less palatable plant biomass (Zhang et al., 2020; Pang et al., 2021) and contributes to degradation of alpine meadows (Liu et al., 2013; Wilson and Smith, 2015). This implies that the contribution of higher disturbance intensity to degradation of alpine meadows is to deteriorate grazing quality of alpine meadows due to low palatable plant biomass (Sun et al., 2015; Wei et al., 2020). Consequently, how to manage plateau pika in alpine meadows is dependent on the operation targets of alpine meadows (Guo et al., 2012). If alpine meadows are operated to exert the ecological and social functions (Guo et al., 2003), the disturbance intensity of plateau pikas can be maintained at a relative higher level. However, alpine meadows are used to graze livestock, plateau pikas had better maintain their low disturbance intensity since palatable plant biomass in alpine meadows firstly increase, than then decrease as the disturbance intensity of plateau pikas increases (Pang and Guo, 2018). Therefore, further studies are necessary to quantify the disturbance intensity threshold of plateau pikas, in which the spatial heterogeneity of vegetation is positive related to palatable plant productivity in alpine meadows.

The data of this study come from three sites that range in elevation from 3,270 to 3,750 m (Table 1) and annual precipitation from 250 to 800 mm, and the results are conclusive. The findings of this study not only indicate that the presence of plateau pikas can lead to higher spatial heterogeneity of vegetation, but also confirms the response of the spatial heterogeneity of vegetation to disturbance intensity of European ground squirrels (Lindtner et al., 2020). Overall, the findings of this study concur with previous findings of the effect of small semi-fossorial herbivores on the spatial heterogeneity of vegetation.

## Conclusion

The plateau pika was employed as an example animal species to investigate the responses of the spatial heterogeneity of vegetation to the presence and the variation in disturbance intensity of a small semi-fossorial mammal across three sites. This study found that the presence of plateau pikas relates to higher spatial heterogeneity of vegetation in alpine meadows. Further, we found a general pattern regarding the effect of disturbance intensity of plateau pika on spatial heterogeneity of vegetation, which can be described by a linear model. These results suggest that the plateau pika is an important biotic factor that alters the spatial heterogeneity of vegetation in alpine meadows. Further, we present a possible approach to estimate the effects of the presence and disturbance intensity of a small semi-fossorial herbivore on the spatial heterogeneity of vegetation in grasslands.

## Data Availability Statement

The original contributions presented in the study are included in the article/supplementary material, further inquiries can be directed to the corresponding author.

## Author Contributions

JL conceived the idea, performed the experiment, analyzed the data, originally drafted, and edited the manuscript. HQ and YD performed the experiment. ZG conceived the idea, found the funding acquisition, supervised the experiment, and polished and edited the manuscript. All authors read and approved the final manuscript.

## Conflict of Interest

The authors declare that the research was conducted in the absence of any commercial or financial relationships that could be construed as a potential conflict of interest.

## Publisher’s Note

All claims expressed in this article are solely those of the authors and do not necessarily represent those of their affiliated organizations, or those of the publisher, the editors and the reviewers. Any product that may be evaluated in this article, or claim that may be made by its manufacturer, is not guaranteed or endorsed by the publisher.
